# Different Levels in Orexin Concentrations and Risk Factors Associated with Higher Orexin Levels: Comparison between Detoxified Opiate and Methamphetamine Addicts in 5 Chinese Cities

**DOI:** 10.1155/2013/282641

**Published:** 2013-09-12

**Authors:** Haoran Zhang, Zhi Lian, Shiyan Yan, Yanping Bao, Zhimin Liu

**Affiliations:** ^1^School of Public Health, Peking University, Beijing 100191, China; ^2^National Institute on Drug Dependence, Peking University, 38 Xueyuan Road Haidian District, Beijing 100191, China; ^3^Institute of Basic Research in Clinical Medicine, Academy of Chinese Medical Sciences, Beijing 100700, China

## Abstract

This study sought to explore the degree of orexin levels in Chinese opiate and methamphetamine addicts and the differences between them. The cross-sectional study was conducted among detoxified drug addicts from Mandatory Detoxification Center (MDC) in five Chinese cities. Orexin levels were assayed with radioimmunoassay (RIA). Mann-Whitney *U* test and Kruskal-Wallis test were used to detect differences across groups, and logistic regression was used to explore the association between orexin levels and characteristics of demographic and drug abuse. Between November 2009 and January 2011, 285 opiates addicts, 112 methamphetamine addicts, and 79 healthy controls were enrolled. At drug withdrawal period, both opiate and methamphetamine addicts had lower median orexin levels than controls, and median orexin levels in opiate addicts were higher than those in methamphetamine addicts (all above *P* < 0.05). Adjusted odds of the above median concentration of orexin were higher for injection than “chasing the dragon” (AOR = 3.1, 95% CI = 1.2–7.9). No significant factors associated with orexin levels of methamphetamine addicts were found. Development of intervention method on orexin system by different administration routes especially for injected opiate addicts at detoxification phase may be significant and was welcome.

## 1. Introduction

Orexins (also named hypocretins) are neuropeptides initially discovered almost simultaneously by de Lecea [1] and Sakurai [2] and are identified as neurotransmitters in lateral hypothalamus (LH) neurons. Since its discovery, studies on orexin have been growing rapidly and made a series of achievements. Many studies have confirmed that the function of orexin neurons was mainly reflected through the regulation of arousal and sleep [3–9], stimulating feeding [10–14], regulating drinking behavior [15], and activating the function of adrenal [16] and cardiovascular system [17]. Later investigations on the orexin system have suggested a possible role of orexins in addictive behaviors [18]. In support of this proposition, a number of research groups began to evaluate the role of orexin on cocaine [19–21], nicotine [22–25], ethanol [26–31], and morphine [11, 32]. For instance, Mori et al. [33] reported that orexin mutant mice have attenuated physical withdrawal from morphine. Li et al. [11] found that after 5 days of treatment (4 mg/kg) twice a day with chronic amphetamine, cAMP-CRMB in orexin neurons was activated. These findings all support the important role of orexin neuropeptides in addiction [28, 31, 34–36].

However, most literatures mainly dealt with that problem based on animal models. Intense research has focused on describing the pathways and mechanisms by which orexins exert their diverse array of functions. To our best knowledge, there were few studies on orexin among illicit drug addicts, especially in China. The aim of this study was to describe and compare orexin levels among Chinese opiates addicts, methamphetamine addicts, and healthy controls and to test whether orexin concentrations were associated with history of drug use by different abused drugs under present data setting.

## 2. Methods

### 2.1. Ethical Statement

The study was approved by the ethical committee of Peking University Health Center (Grant number IRB00001052-10026). Before the interviews and blood sampling were conducted, subjects were informed about the purpose of this study and confidentiality of all information they would provide. Respondents' consent was gathered. The survey was conducted in an isolated room, and the survey procedures were designed to protect privacy of subjects by allowing for anonymous and voluntary participation. Participants in the study could stop at any time if they desired.

### 2.2. Site and Population

A multicenter survey was conducted from the Mandatory Detoxification Center (MDC) in 5 cities, including Beijing, Guangzhou (Guangdong Province), Shenzhen (Guangdong Province), Taiyuan (Shanxi Province), and Xi'an (Shanxi Province). The research locations were chosen for practical reasons. Beijing, Guangzhou, and Shenzhen are metropolitan and most densely populated in China. Taiyuan and Xi'an city were capital cities and smaller in size. Each city represents different geographic regions including northland, midland, southland and westland urban regions of China. Moreover, MDC is administered by the Ministry of Public Security, and for repeat drug offenders, the Ministry of Public Security does not provide drug replacement therapy to addicts [37], which helped facilitate organization and recruitment of participants.

Participants were recruited using cluster sampling. The eligibility criteria were (1) meet the (Diagnostic and Statistical Manual of Mental Disorders, four edition (DSM-IV); American Psychiatric Association, 1994b) criteria for opiate dependence and methamphetamine dependence; (2) men aged 18 or over; (3) for opiate addicts, predominate drugs of choice were only opiates; and for methamphetamine addicts, predominate drugs of choice were only methamphetamine in the baseline survey; (4) received mandatory detoxification treatment for less than 12 months; (5) willing to sign an informed consent; and (6) adhered sufficiently to the study protocol. Meanwhile, the exclusionary criteria were made: (1) unwilling to participate or could not provide written informed consent; (2) could not discriminate the primary drug of abuse; (3) severe psychotic disorders; (4) severe body diseases (e.g., prostrate disease of liver or kidney); (5) be apt to drop out; and (6) deficiency in or lack of language and comprehensive problems. For better understanding, men from community were selected as healthy controls. The criteria were (1) aged 18 and above; (2) without a history of drug abuse before the study and no evidence of drug abuse during study; and (3) provided informed consent. 

Based on registration information of MDC, samples of drug addicts were stratified into opiate and methamphetamine addicts. It is necessary to explain that opiates are a category of drugs including heroin and morphine. Within this study, heroin was the main opiate used by respondents. In addition, participants did not simultaneously use both opiates and methamphetamine. Polydrug users were excluded from this study. Overall, a total of 476 eligible subjects were recruited between November 2009 and January 2011. The sample of analysis included 285 opiates addicts, 112 methamphetamine addicts, and 79 healthy men.

### 2.3. Characterization of Demographics and History of Drug Use

 The baseline survey by self-administered questionnaire included age of subjects, ethnicity, marital status, regular dosage by gram each time, months of drug abuse, and routes of drug abuse. The survey period was set from initiation phase to enter MDC. Responses for opiates and methamphetamine use on administration routes were chasing the dragon (which refers to inhaling the vapor from heated morphine, heroin, or opium that has been placed on a piece of foil), intravenous injection, intramuscular injection, smoking in a cigarette, snorting, and other routes. Due to sparseness of data and lack of meaningful differences in some routes of administration, intravenous injection and intramuscular injection were grouped into category of injection. Smoking a cigarette, snorting, and other routes were classified as others. Therefore, the response options for administration routes were defined as chasing the dragon, injection, and others.

### 2.4. Measurement of Orexin Concentration

 In view of circadian rhythm of orexin hormone secretion and in order to avoid the influence of food, therefore, the collection of blood specimen was fixed before breakfast. All drug abusers were requested to collect blood specimen with a limosis status. Five milliliter (mL) blood samples were obtained by venipuncture and immediately cooled on ice. Plasma was separated by centrifugation with 4000 rpm for 8 minutes and then stored at below 20 centigrade. The assays were performed in one batch as soon as the study was completed. To measure orexin, radioimmunoassay (RIA) with reagent box (produced by Xinwan Biology and Science Inc., Tianjin, China) was used. And intra-assay coefficients of variation were all less than 10.0% and interassay coefficients of variation were all less than 15.0%.

### 2.5. Study Design and Procedures

The cross-sectional survey was employed in this study. Each investigation included an administered questionnaire and a specimen. In order to eliminate and prevent possible bias, a pilot trial was carried out. At the beginning of formal survey, the experienced research staff explained the purpose of this study. Investigation was conducted in private and undisturbed rooms. All finished questionnaires were reviewed by researchers for completeness and consistency. Following the survey, respondents were asked to provide blood specimen for orexin testing. The process would take about 30 min for each subject. 

### 2.6. Data Analyses

As a result of orexin concentrations were not normally distributed; the median with interquartile range was used to present the skewed distributions of this variable. The differences of orexin concentrations in numerical variables were compared using Kruskal-Wallis test. Where necessary, Mann-Whitney *U* test was carried out. The chi-square test was used in qualitative variables. To understand factors related to orexin levels, the outcome variables were recoded into two categories (if higher than the median level of orexin as dependent variables; higher than or equal to the median level = 1; lower than the median level = 0). The age of subjects (≤35 years and >35 years), regular median dosage (≤0.5 and >0.5) (g/each time), and months of drug use (≤12 and >12 month) were categorized into two categories, respectively. The factors associated with orexin levels were analyzed using binary logistic regression models. If possible, multiple logistic regression was used to explore the related factors after adjustment. Data were entered twice with Epi-Data software and then validated until two inputs were completely the same. Statistical analyses were conducted using SPSS. A two-tailed alpha for statistical difference was set at 0.05.

## 3. Results

### 3.1. Characteristics of Demographic and History of Drug Use

Demographic characteristics and history of drug use were summarized in Table 1. At baseline, significant difference was observed in respondent's average age (*F* = 16.280, *P* < 0.001) among three groups. There were significant differences in ethnicity (*χ* ^2^ = 23.935, *P* < 0.001), marital status (*χ* ^2^ = 42.145, *P* < 0.001), median months of drug use (Mann-Whitney *U* = 6782.000, *P* < 0.001), and administration routes (*χ* ^2^ = 23.448, *P* < 0.001) between opiate and methamphetamine groups, respectively. No statistical difference was found between opiate and methamphetamine groups in regard to median dosage of drug use (Mann-Whitney *U* = 8635.500, *P* = 0.078).

### 3.2. Comparison of Orexin Levels among Opiates, Methamphetamine, and Healthy Groups

**Figure 1:**
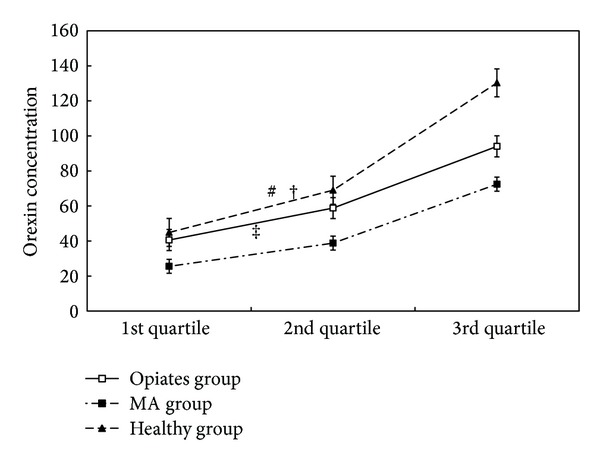
Quartiles of orexin median concentrations (pg/mL) in opiate group, MA group, and healthy group. *Notes*. Statistical significance between groups was set at 0.0167 in order for type I error levels to be guaranteed. Significant differences of orexin concentrations for opiates, methamphetamine (MA), and healthy groups were found by Kruskal-Wallis test (*χ* ^2^ = 32.690, *P* < 0.001). ^#^ *P* > 0.0167 in comparison with the median of healthy group (Mann-Whitney *U* = 9472.000, *P* = 0.031). ^†^ *P* < 0.0167 in comparison with the median of MA group (Mann-Whitney *U* = 10956.500, *P* < 0.001). ^‡^ *P* < 0.0167 in comparison with the median of healthy group (Mann-Whitney *U* = 2595.000, *P* < 0.001).

Figure 1 detailed the median with interquartile range concentrations of orexin in the drug addicts and controls. In total, Kruskal-Wallis test showed a significant difference in median orexin concentrations among opiate, methamphetamine, and healthy groups (*χ* ^2^ = 32.690, *P* < 0.001). For opiate and methamphetamine addicts, both of them had lower median orexin levels than healthy controls. Specifically, the median concentration of orexin in methamphetamine addicts was lower than that in healthy males (Mann-Whitney *U* = 2595.000, *P* < 0.001). However, Mann-Whitney *U* test just failed to show a significant difference in median orexin levels between opiate and healthy groups (Mann-Whitney *U* = 9472.000, *P* = 0.031). Otherwise, the median concentration of orexin in opiate addicts was higher than that in methamphetamine addicts (Mann-Whitney *U* = 10956.500, *P* < 0.001). 

### 3.3. Factors Associated with Orexin Levels in Opiate and Methamphetamine Addicts

**Figure 2:**
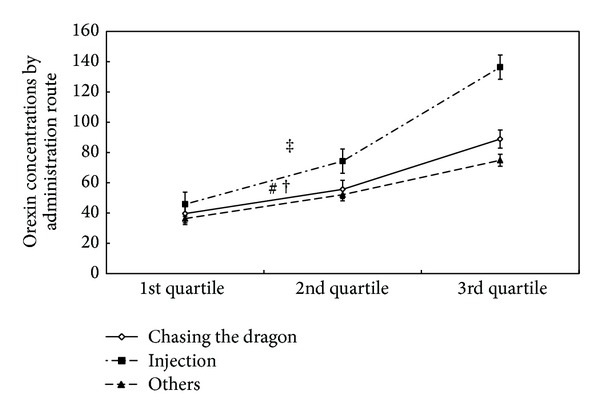
Quartiles of orexin median concentrations (pg/mL) by administration routes in opiate addicts. In total, significant differences of orexin concentrations for chasing the dragon, injection, and other routes of drug administration were found by Kruskal-Wallis test (*χ* ^2^ = 10.201, *P* < 0.01). As a result of that, *α* = 0.0167 was used when tests were calculated between any two groups. ^#^No significant difference in comparison with other routes of drug administration (Mann-Whitney *U* = 1991.500, *P* = 0.332). ^†^Significantly different from administration route of injection (Mann-Whitney *U* = 5577.000, *P* = 0.005). ^‡^Significantly different from other routes of drug administration (Mann-Whitney *U* = 661.000, *P* = 0.013).

Kruskal-Wallis test found significant differences between administration routes and the median of orexin concentrations (*χ* ^2^ = 10.201, *P* < 0.01) (Figure 2). The median level of orexin for injection administration (74.29 pg/mL) was approximately 19 pg/mL higher than chasing the dragon (55.61 pg/mL) (Mann-Whitney *U* = 5577.000, *P* < 0.0167). However, no significant differences were observed between months, regular dosage of drug use, and the median orexin concentrations. The binary logistic regression analysis showed that other routes of administration were more likely to have higher median of orexin concentrations than chasing the dragon (Odds Ratio, OR = 2.904, *P* < 0.05) (Table 2). Although injection drug users tended to report higher median levels than users choosing chasing the dragon method, this effect did not reach statistical significance (OR = 1.609, *P* > 0.05). Using the above median concentration of orexin as the referent, the multiple logistic regression showed that the adjusted odds were higher for injection than chasing the dragon (OR = 3.1, 95% CI = 1.2–7.9) (Table 3). The adjusted variables included age, marital status, months of drug use, and regular administrated dosage.

## 4. Discussion

The present study showed that both opiate and methamphetamine (MA) addicts had lower orexin levels than healthy controls, and orexin levels in opiate addicts were higher than those in methamphetamine addicts. To our knowledge, this comparison was not found in other studies. For opiate addicts, route of injection was associated with higher levels of orexin. But no association was found between orexin levels and demographic characteristics and drug abuse history for methamphetamine addicts.

In this study, we found that both opiate and methamphetamine addicts had lower median orexin levels than healthy men. Firstly, the served mandatory detoxification may have partly contributed to this effect. Some previous studies have found that stress-induced reinstatement was critically dependent on the drug withdrawal period [38, 39]. Wang et al. [40] found ventral tegmental area (VTA) perfusion of orexin stress reinstated cocaine seeking, which demonstrated that orexin was involved in the process of stress-induced relapse. Withdrawal duration may be an important stress factor for inhibiting orexin. However, this hypothesis needs support from comparison among detoxified addicts in different withdrawal period. Data were collected only for less than 12 months of detoxification in this survey, for those who served more than 12 months, if orexin levels would restore to normal compared to healthy controls needs further study. Secondly, it was reasoned that orexin receptors in the lateral hypothalamus (LH) or the mesolimbic dopamine system were inhibited [34, 41] during the initial detoxification, which expressed itself as the inhibited desire for drug seeking. For example, Narita et al. [42] suggested that the excitation of brain orexin neurons and/or the tonic activation of orexin receptors were specifically required for the rewarding effect of opiates.

Data here also suggest higher median levels of orexin in opiate addicts than in methamphetamine addicts. The underlying reasons may be the following: effect of abused drugs on the orexin system was drug-specific [24], and regional variations within the LH have been reported in response to some drugs. For instance, Fos expression was increased in orexin neurons following acute administration of methamphetamine [37], and acute morphine fails to alter orexin mRNA levels [43]. However, it could not provide more information about the significance of this comparison, because the pharmacological, cytological, and neurobiological data were not obtained in the current investigation. Notwithstanding, it led us to assume that the degree of inhibited orexin system in opiates addicts was slighter than that in methamphetamine addicts, which beget opiate addicts were more liable to rehabilitation.

This study found that orexin levels for opiate addicts differed by administration routes (*P* = 0.033) and injected opiate addicts had higher median orexin levels. This may be supported by the finding that injection administration was associated with the above median levels of orexin among opiate addicts (AOR = 3.076, 95% CI: 1.194–7.909). Previous studies have shown that routes of drug administration differed significantly by drugs of abuse [44]. We speculate that administration routes affect the orexin levels of opiate addicts, because injected opiate appeared to activate orexin neurons in this population. Similarly, Harris et al. [45] found that injected morphine could stimulate LH orexin neurons during conditioned place preference (CPP). However, the underlying mechanism between administration routes and orexin levels still needs further examination.

Limitations of this study should be discussed. Although it has been shown that central and peripheral orexin levels increase and decrease in similar proportions, the first limitation of this study may be attributed to the peripheral analysis of orexin levels as a centrally generated metabolite [46]. Second, this study could not neglect the effect of demographic characters, owing to the fact that significant differences between groups were observed. We had tried to match confounding factors such as age and marital status. Thus, demographic characteristics were used as a control in real analysis. Moreover, female participants were precluded, but a finding [47] showed that female injected drug users (IDUs) in Tijuana had an even higher prevalence of methamphetamine (MA) use compared to males which warrant exploration in future studies. Third, in spite of many efforts to increase the sample size of blood specimen, the overall number of MA and healthy controls remained little. To some extent, it may not meet for sufficient power to detect significant differences in logistic analysis. Larger scale survey is needed to provide detailed information and extend present findings. Last but not least, comparison of orexin levels before and after detoxification was not executed due to lack of related data, which more or less limited the interpretation of results.

## 5. Conclusions

Despite these limitations, as far as we know, less was known about the levels and influenced factors of orexin concentrations among different groups in China. From a clinical point of view, the discovery of the linkage between orexin levels and injection administration may lead to the development of a special detoxified means for opiate addicts and to the expectation of the ultimate development of novel drugs for treatment of opiate addicts.

## Figures and Tables

**Table 1:** Demographic characteristics and history of drug use among three different groups.

Variables	Opiates (*N* = 285)	MA* (*N* = 112)	Healthy^‡^ (*N* = 79)
Demographic characteristics			
Age (year) (valid total^†^)	36.4 ± 8.2 (285)	31.3 ± 7.6 (106)	32.8 ± 9.8 (79)
Han ethnicity (%) (valid total^†^)	76.8 (285)	51.8 (112)	—
Married (%) (valid total^†^)	54.7 (285)	18.8 (112)	—
History of drug use			
Median months of drug abuse (valid total^†^)	7.7 (280)	12.0 (71)	—
Regular median dosage (g/each time) (valid total^†^)	0.4 (277)	0.2 (72)	—
Chasing the dragon versus injection (%) (valid total^†^)	63.5 (181) versus 27.7 (79)	51.8 (58) versus 0	—

^†^Total number may not be equal to the number of cases due to excluding missing data.

^‡^Data of healthy group were collected only for age of cases.

*Methamphetamine.

**Table 2:** Unadjusted odds ratios and 95% confidence interval for the relationship between a concentration above the median of the orexin (pg/mL) and demographic characteristics and history of drug use among different groups.

Variables	Opiate group				MA* group			
	*N *	Median (IQR^†^)	*P *	OR (95% CI)	*N *	Median (IQR^†^)	*P *	OR (95% CI)
Age (year)								
≤35	119	58.13 (40.89–87.78)	0.787	0.937 (0.585–1.501)	78	42.42 (28.06–77.70)	0.153	1
>35	166	60.24 (39.98–103.14)		1	28	31.90 (23.04–58.78)		1.899 (0.788–4.577)
Regular median dosage (g/each time)								
≤0.5	196	56.92 (39.20–93.73)	0.303	1	63	40.47 (25.14–92.00)	0.342	1
>0.5	89	62.08 (41.55–95.38)		0.768 (0.464–1.270)	49	36.33 (25.96–50.81)		1.439 (0.680–3.046)
Routes of administration at addiction phase								
Chasing the dragon	181	55.61 (39.60–88.87)	0.033	1	58	43.09 (29.56–77.70)	0.450	1
Injection	79	74.29 (45.80–136.40)		1.609 (0.676–3.831)	—	—		—
Others	25	52.07 (36.34–74.86)		2.904 (1.140–7.393)	54	36.33 (24.50–62.00)		0.751 (0.357–1.579)
Months of drug use								
≤12	210	59.87 (40.73–94.51)	0.610	1	36	43.31 (24.66–90.76)	0.419	1
>12	75	57.72 (40.04–92.73)		0.872 (0.514–1.478)	76	37.14 (26.44–62.37)		1.389 (0.626–3.081)

Exposure was defined as the above median concentration of orexin.

^†^IQR; interquartile range.

*Methamphetamine.

**Table 3:** Multiple logistic regression for associations between administration routes of drug use and a concentration above the median of the orexin (pg/mL) among opiate addicts.

Administration routes of drug use	*N *	Adjusted OR	95% CI	*P *
Chasing the dragon	181	1		
Injection	79	3.073	1.194–7.909	0.020
Others	25	1.720	0.687–4.304	0.247

Adjusted for age of subjects, marital status, months of drug use, and dosage of drug use.

## References

[1] Pasumarthi RK, Fadel J (2010). Stimulation of lateral hypothalamic glutamate and acetylcholine efflux by nicotine: implications for mechanisms of nicotine-induced activation of orexin neurons. *Journal of Neurochemistry*.

[2] Wisor JP, Nishino S, Sora I, Uhl GH, Mignot E, Edgar DM (2001). Dopaminergic role in stimulant-induced wakefulness. *Journal of Neuroscience*.

[3] Taheri S, Hafizi S (2002). The orexins/hypocretins: hypothalamic peptides linked to sleep and appetite. *Psychological Medicine*.

[4] Yoshida Y, Fujiki N, Maki RA, Schwarz D, Nishino S (2003). Differential kinetics of hypocretins in the cerebrospinal fluid after intracerebroventricular administration in rats. *Neuroscience Letters*.

[5] Selbach O, Eriksson KS, Haas HL (2003). Drugs to interfere with orexins (Hypocretins). *Drug News and Perspectives*.

[6] Ishibashi M, Takano S, Yanagida H (2005). Effects of orexins/hypocretins on neuronal activity in the paraventricular nucleus of the thalamus in rats in vitro. *Peptides*.

[7] Tao R, Ma Z, McKenna JT (2006). Differential effect of orexins (hypocretins) on serotonin release in the dorsal and median raphe nuclei of freely behaving rats. *Neuroscience*.

[8] Carter ME, Borg JS, de Lecea L (2009). The brain hypocretins and their receptors: mediators of allostatic arousal. *Current Opinion in Pharmacology*.

[9] Eriksson KS, Sergeeva OA, Haas HL, Selbach O (2010). Orexins/hypocretins and aminergic systems. *Acta Physiologica*.

[10] Kenny PJ (2011). Tobacco dependence, the insular cortex and the hypocretin connection. *Pharmacology Biochemistry and Behavior*.

[11] Li Y, Wang H, Qi K (2011). Orexins in the midline thalamus are involved in the expression of conditioned place aversion to morphine withdrawal. *Physiology and Behavior*.

[12] Von der Goltz C, Koopmann A, Dinter C (2011). Involvement of orexin in the regulation of stress, depression and reward in alcohol dependence. *Hormones and Behavior*.

[13] De Lecea L, Sutcliffe JG (1999). The hypocretins/orexins: novel hypothalamic neuropeptides involved in different physiologicaI systems. *Cellular and Molecular Life Sciences*.

[14] Taheri S, Bloom S (2001). Orexins/hypocretins: waking up the scientific world. *Clinical Endocrinology*.

[15] Li S-X, Liu L-J, Jiang W-G (2010). Circadian alteration in neurobiology during protracted opiate withdrawal in rats. *Journal of Neurochemistry*.

[16] Taheri S, Zeitzer JM, Mignot E (2002). The role of hypocretins (Orexins) in sleep regulation and narcolepsy. *Annual Review of Neuroscience*.

[17] López M, Tena-Sempere M, Diéguez C (2010). Cross-talk between orexins (hypocretins) and the neuroendocrine axes (hypothalamic-pituitary axes). *Frontiers in Neuroendocrinology*.

[18] Bonini JA, Jones KA, Adham N (2000). Identification and characterization of two G protein-coupled receptors for neuropeptide FF. *Journal of Biological Chemistry*.

[19] Zhang G-C, Mao L-M, Liu X-Y, Wang JQ (2007). Long-lasting up-regulation of orexin receptor type 2 protein levels in the rat nucleus accumbens after chronic cocaine administration. *Journal of Neurochemistry*.

[20] Smith RJ, See RE, Aston-Jones G (2009). Orexin/hypocretin signaling at the orexin 1 receptor regulates cue-elicited cocaine-seeking. *European Journal of Neuroscience*.

[21] Smith RJ, Tahsili-Fahadan P, Aston-Jones G (2010). Orexin/hypocretin is necessary for context-driven cocaine-seeking. *Neuropharmacology*.

[22] Hollander JA, Lu Q, Cameron MD, Kamenecka TM, Kenny PJ (2008). Insular hypocretin transmission regulates nicotine reward. *Proceedings of the National Academy of Sciences of the United States of America*.

[23] Plaza-Zabala A, Martín-García E, De Lecea L, Maldonado R, Berrendero F (2010). Hypocretins regulate the anxiogenic-like effects of nicotine and induce reinstatement of nicotine-seeking behavior. *Journal of Neuroscience*.

[24] Sharf R, Sarhan M, DiLeone RJ (2010). Role of orexin/hypocretin in dependence and addiction. *Brain Research*.

[25] Bonci A, Borgland S (2009). Role of orexin/hypocretin and CRF in the formation of drug-dependent synaptic plasticity in the mesolimbic system. *Neuropharmacology*.

[26] Richards JK, Simms JA, Steensland P (2008). Inhibition of orexin-1/hypocretin-1 receptors inhibits yohimbine-induced reinstatement of ethanol and sucrose seeking in Long-Evans rats. *Psychopharmacology*.

[27] Cannella N, Economidou D, Kallupi M (2009). Persistent increase of alcohol-seeking evoked by neuropeptide S: an effect mediated by the Hypothalamic Hypocretin System. *Neuropsychopharmacology*.

[28] Jupp B, Krstew E, Dezsi G, Lawrence AJ (2011). Discrete cue-conditioned alcohol-seeking after protracted abstinence: pattern of neural activation and involvement of orexin1 receptors. *British Journal of Pharmacology*.

[29] Shoblock JR, Welty N, Aluisio L (2011). Selective blockade of the orexin-2 receptor attenuates ethanol self-administration, place preference, and reinstatement. *Psychopharmacology*.

[30] Quarta D, Valerio E, Hutcheson DM, Hedou G, Heidbreder C (2010). The orexin-1 receptor antagonist SB-334867 reduces amphetamine-evoked dopamine outflow in the shell of the nucleus accumbens and decreases the expression of amphetamine sensitization. *Neurochemistry International*.

[31] Rudzińska U, Zalewska-Kaszubska J (2009). Role of endogenous neuropeptides in the pathomechanism of alcohol addiction. *Postpy Higieny i Medycyny Doświadczalnej*.

[32] Azizi H, Mirnajafi-Zadeh J, Rohampour K, Semnanian S (2010). Antagonism of orexin type 1 receptors in the locus coeruleus attenuates signs of naloxone-precipitated morphine withdrawal in rats. *Neuroscience Letters*.

[33] Mori T, Ito S, Kuwaki T, Yanagisawa M, Sakurai T, Sawaguchi T (2010). Monoaminergic neuronal changes in orexin deficient mice. *Neuropharmacology*.

[34] James MH, Charnley JL, Levi EM (2011). Orexin-1 receptor signalling within the ventral tegmental area, but not the paraventricular thalamus, is critical to regulating cue-induced reinstatement of cocaine-seeking. *International Journal of Neuropsychopharmacology*.

[35] Borgland SL, Ungless MA, Bonci A (2010). Convergent actions of orexin/hypocretin and CRF on dopamine neurons: emerging players in addiction. *Brain Research*.

[36] Boutrel B, Cannella N, de Lecea L (2010). The role of hypocretin in driving arousal and goal-oriented behaviors. *Brain Research*.

[37] Estabrooke IV, McCarthy MT, Ko E (2001). Fos expression in orexin neurons varies with behavioral state. *Journal of Neuroscience*.

[38] Shalev U, Grimm JW, Shaham Y (2002). Neurobiology of relapse to heroin and cocaine seeking: a review. *Pharmacological Reviews*.

[39] Han J, Li YH, Bai YJ (2007). Role of hypothalamic orexin in drug addiction. *Sheng Li Ke Xue Jin Zhan*.

[40] Wang B, You Z-B, Wise RA (2009). Reinstatement of cocaine seeking by hypocretin (Orexin) in the ventral tegmental area: independence from the Local Corticotropin-Releasing Factor Network. *Biological Psychiatry*.

[41] Borgland SL, Taha SA, Sarti F, Fields HL, Bonci A (2006). Orexin a in the VTA is critical for the induction of synaptic plasticity and behavioral sensitization to cocaine. *Neuron*.

[42] Narita M, Nagumo Y, Hashimoto S (2006). Direct involvement of orexinergic systems in the activation of the mesolimbic dopamine pathway and related behaviors induced by morphine. *Journal of Neuroscience*.

[43] Zhou Y, Bendor J, Hofmann L, Randesi M, Ho A, Kreek MJ (2006). Mu opioid receptor and orexin/hypocretin mRNA levels in the lateral hypothalamus and striatum are enhanced by morphine withdrawal. *Journal of Endocrinology*.

[44] Strang J, Bearn J, Farrell M (1998). Route of drug use and its implications for drug effect, risk of dependence and health consequences. *Drug and Alcohol Review*.

[45] Harris GC, Wimmer M, Randall-Thompson JF, Aston-Jones G (2007). Lateral hypothalamic orexin neurons are critically involved in learning to associate an environment with morphine reward. *Behavioural Brain Research*.

[46] von der Goltz C, Koopmann A, Dinter C (2010). Orexin and leptin are associated with nicotine craving: a link between smoking, appetite and reward. *Psychoneuroendocrinology*.

[47] Rusch ML, Lozada R, Pollini RA (2009). Polydrug use among IDUs in Tijuana, Mexico: correlates of methamphetamine use and route of administration by gender. *Journal of Urban Health*.

